# Fecobionics in proctology: review and perspectives

**DOI:** 10.1016/j.soda.2023.100117

**Published:** 2023-11-28

**Authors:** H. Gregersen, D. Sun, F. Field, W. Combs, P. Christensen, H. Mousa, F.J. Moawad, S. Eisenstein, G.S. Kassab

**Affiliations:** a California Medical Innovations Institute, San Diego, California, USA; b School of Microelectronics and Communication Engineering, Chongqing University, Chongqing, China; c S3DT Holdings, San Diego, California, USA; d Department of Surgery, Aarhus University Hospital, Aarhus, Denmark; e CHOP, University of Pennsylvania, Philadelphia, Pennsylvania; f Scripps Clinic, Division of Gastroenterology, La Jolla, California, USA; g Department of Surgery, University of California San Diego, La Jolla, California, USA

**Keywords:** Anorectal function, Defecation, Fecal incontinence, Fecobionics, Obstructed defecation, Simulated stool

## Abstract

Fecobionics is a novel integrated technology for assessment of anorectal function. It is a defecatory test with simultaneous measurements of pressures, orientation, and device angle (a proxy of the anorectal angle). Furthermore, the latest Fecobionics prototypes measure diameters (shape) using impedance planimetry during evacuation of the device. The simultaneous measurement of multiple variables in the integrated test allows new metrics to be developed including more advanced novel defecation indices, enabling mechanistic insight in the defecation process at an unprecedented level in patients with anorectal disorders including patients suffering from obstructed defecation, fecal incontinence, and low anterior resection syndrome. The device has the consistency and shape of a normal stool (type 3–4 on the Bristol Stool Form Scale). Fecobionics has been validated on the bench and in animal studies and used in clinical trials to study defecation phenotypes in normal human subjects and patients with obstructed defecation, fecal incontinence, and low anterior resection syndrome after rectal cancer surgery. Subtypes have been defined, especially of patients with obstructed defecation. Furthermore, Fecobionics has been used to monitor biofeedback therapy in patients with fecal incontinence to predict the outcome of the therapy (responder versus non-responder). Most Fecobionics studies showed a closer correlation to symptoms as compared to current technologies for anorectal assessment. The present article outlines previous and ongoing work, and perspectives for future studies in proctology, including in physiological assessment of function, diagnostics, monitoring of therapy, and as a tool for biofeedback therapy.

## Introduction

The gastrointestinal (GI) tract including the anorectum has traditionally presented challenges for studies of its physiological function and for diagnostics. The anorectal (defecatory) function is complex, a fact that is reflected in the high numbers of patients with symptoms arising from the anorectum [7,16,19,35,46,52]. Anal continence and defecation involve anatomical factors, anorectal sensation, rectal compliance, stool consistency, anal muscle strength, mobility, and psychological factors [4,26,48]. Hence, the major mechanical determinants of defecation are the fecal volume, the rectoanal pressure gradient, anal diameter, and the anorectal angle.

The homeostatic balance in the defecation process is easily disturbed and altered in disease conditions, resulting in symptoms such as pain (proctalgia), fecal incontinence (FI) and constipation [5,48,56]. Chronic constipation is often caused by obstructed defecation (OD), which may be due to dyssynergia [51]. Anorectal sensitivity and contractility, stool consistency, rectal reservoir capacity and compliance, and coordination of the pelvic floor muscles play an important role in the genesis of OD. Defecatory disorders affect 20 % of the population [3,4,35,46,48,52]. In contrast, FI is a symptom associated with the lack of control of bowel movements, i.e., fecal material leaks from the rectum without warning, or with a strong urge to defecate which cannot be withheld. FI has underlying causes such as anal sphincter incompetence or rectal hypersensitivity. Like OD, FI poses a major health care burden but is poorly recognized and treated [48].

Knowledge of the defecation process in health and disease has steadily increased over the past decades due to technologies such as high-resolution anorectal manometry (HRAM, [8,13,57]), balloon expulsion technology (BET) [8,12,45,47,61], barium and MRI defecography [5,48,59] and the less commonly used Functional Luminal Imaging Probe (FLIP), electromyography, anal ultrasonography, and the barostat [1,5,42,48]. Collectively, these technologies measure important variables that are related to the outcome of the defecation process, including pressures (a proxy of propulsive and resistive forces), diameters (shape), and the anorectal angle along with sensation.

Despite significant developments in the field, some of the current tests have limitations, e.g., barium and MR defecography do not provide direct information on the anorectal sensory and motor function. BET only measures the duration of the evacuation of the balloon. HRAM does not record dynamic events during evacuations but instead simulates defecation by the push procedure. They tests measure different aspects of anorectal function, e.g., HRAM measures anal pressure whereas FLIP measures anal distensibility. An additional major limitation is that these tests are done separately. Therefore, it is not surprising that there is considerable disagreement between the results of anorectal tests and that they correlate poorly with the patient’s symptoms and treatment outcomes [32,45]. The discrepancy between tests is recognized in the Rome criteria for anorectal disorders, where it is not required that all tests show abnormality for a given disorder. It is disadvantageous, however, to have standardization criteria built on discrepancy between tests.

Due to the unmet need for innovation in this field, a novel Fecobionics technology was presented in 2017 that integrates most measurements of currents tests and beyond [21]. Fecobionics is a highly advanced simulated stool with the shape and consistency of a normal stool (corresponds to type 3–4 on the Bristol Stool Form Scale (BSFS). Types 3–4 are found in +60 % of healthy subjects [38]. Fecobionics records pressures, orientation, acceleration, bending (anorectal angle) and shape changes during attempted evacuation of the device. All measurements are recorded in a single experiment under the same conditions, providing the impetus for calculation of unprecedented novel metrics. As outlined in more detail below, Fecobionics has been used to study defecation patterns in normal subjects and patients with OD, FI, and low anterior resection syndrome (LARS) after rectal cancer surgery [10,11,21,23–25,27,39,54,55]. Furthermore, the technology has been used to monitor biofeedback therapy in FI patients [17]. Precise diagnostics and monitoring are important for optimal therapy including monitoring of the effect of dietary changes, drugs, biofeedback therapy, and sacral nerve stimulation.

The aim of this article is to review previous and ongoing Fecobionics work and to provide perspectives for future clinical studies including in pediatrics and in the surgical field.

### Principle of Fecobionics measurements

Fecobionics is a simulated stool capable of dynamic integrated measurements in the lower GI tract [21,24,27,39,54,55]. It was developed as a simulated stool that integrates several current technologies in a single examination. Basically, two comparable devices have been developed. The first version originating in 2017 were wired, contained pressure sensors, and had the ability to measure bending, a proxy of the anorectal angle during evacuation of the device (Gregersen et al. 2017). The second version was wireless, i.e., with internal batteries and wireless transmitter [29]. It had higher quality electronic components embedded, improved algorithms for bending analysis, implemented impedance planimetric measurement of luminal cross-sectional area (CSA) or diameters, and had a much-improved graphical user interface.

**Fig. 1.**
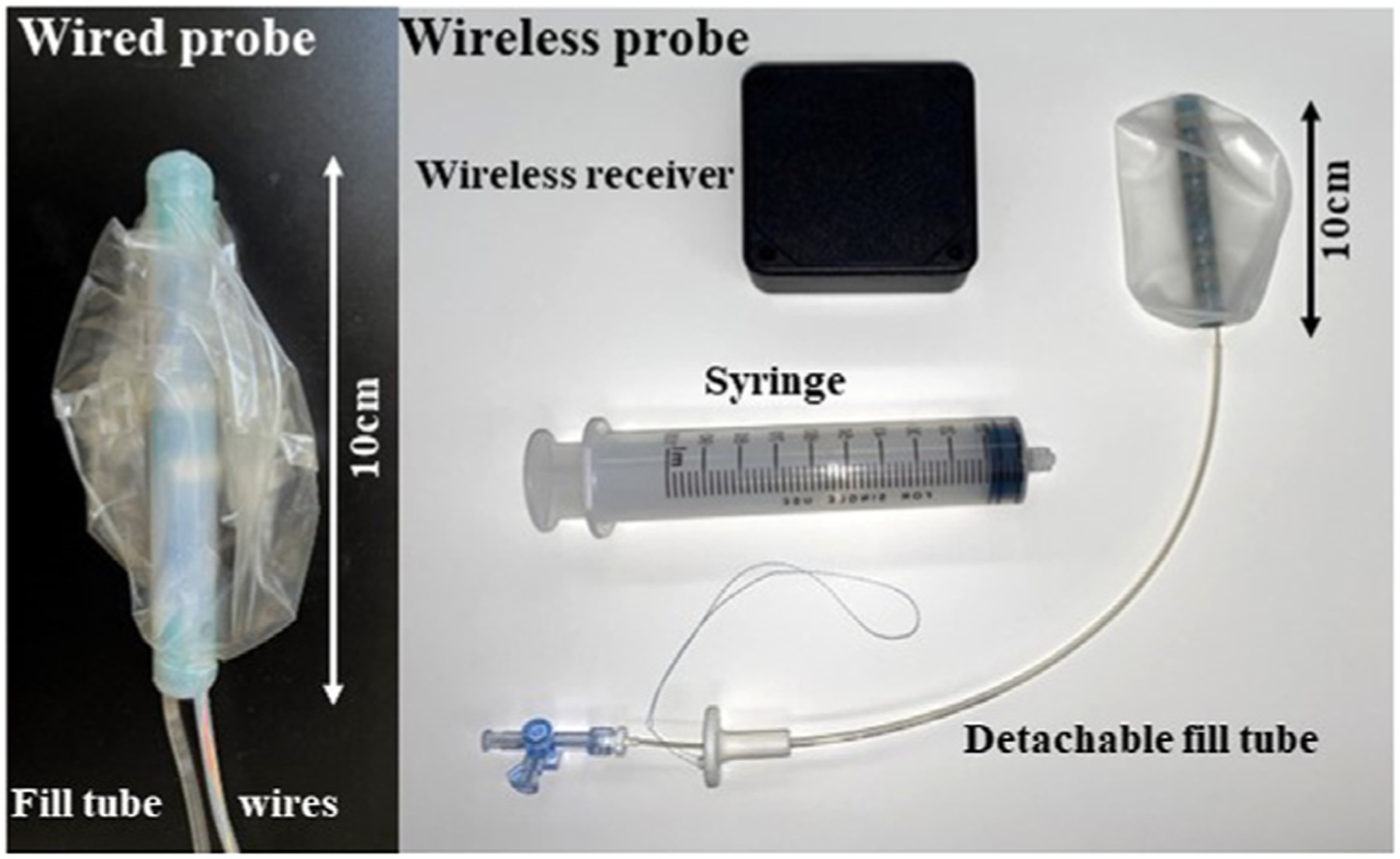
The wired and the wireless Fecobionics devices. **Left:** The wired probe contains pressure sensors and motion processor units for measurement of orientation and bending. It connects to the USB port of a computer through wires inside a thin tube. Furthermore, it contains a thin tube for filling the bag. **Right:** The wireless Fecobionics with pressure sensors, motion processor units, impedance electrodes, CPU, wireless transmitter, and internal batteries. The fill tube is detachable, meaning that the device is untethered after rectal distension and removal of the tube. The probe transmits data in real time to a wireless receiver hub that further transmits the data to a laptop.

The basic design of Fecobionics has been described [24,29,54]. Photos of the wired and wireless Fecobionics devices are shown in Fig. 1. In brief, Fecobionics is an elongated flexible probe (10cm-long, 10–12 mm diameter) made from medical grade silicone rubber with embedded electronics. Silicone is the ideal material for the core due to its softness (bendability), durability, non-degradability, medical use, and electrical current insulation. The silicone core insulates the electrical components from being in direct contact with tissue, which is important if batteries leak, or the electronics short-circuits. The core is very bendable to avoid that the device properties affect the measurement of the anorectal angle.

The Fecobionics technology contains the following sensors:

- Pressure sensors at the front, middle (inside the bag), and at the rear. The unique feature of Fecobionics is that the front and rear pressure measurements are in axial (longitudinal) direction, i.e., in the direction of its trajectory during evacuation. This contrasts with catheter-based manometry systems that measure radial pressures. The implication of axial pressure measurements is that they correlate with the propulsive and resistive forces during evacuation.
- Motion processor units (MPUs) at the front and rear of the device. Each MPU contains 3D gyroscopes, accelerometers, and magnetometers. Hence, the orientation of the device, its acceleration, velocity of expulsion and position can be measured or computed. Furthermore, by using a modification of the Madgwick algorithm [43], the bending of the device is computed independent of the orientation and direction of the bending. The original wired Fecobionics device used 6-axis MPUs whereas the newest wireless device embedded 9-axis MPUs.
- Impedance electrodes on the surface of the core inside the bag (only in the newest Fecobionics device) that measure seven cross-sectional areas (CSAs) along the length of the device. Using the impedance planimetric principle [2,22,28,30], as also used in the functional luminal imaging technology (FLIP) [42], two outer electrodes generate a constant alternating electrical field inside the saline-filled distension bag. Multiple equidistant detection electrodes measure the impedance of the fluid between them, which can be calibrated to diameters or CSAs. The bag shape can be computed from the serial CSAs. Furthermore, CSA changes can be used to determine the velocity of expulsion when the device passes the narrow anal canal.

Filling of the bag is not only done to measure CSAs but also for rectal sensitivity testing. The bag spans most of the length of the device core and can contain volumes up to 120 ml. The distension is stopped at lower volumes, however, where the subject feels urge-to-defecate. The bag is connected through a thin tube extending from the front of Fecobionics to a syringe containing saline. In the latest Fecobionics device, the filling tube is detachable, i.e., after filling the bag inside rectum to the urge-to-defecate level, the tube can be detached from the device and removed. This leaves Fecobionics untethered during the evacuation that happens in privacy without the investigators in the room. The wired Fecobionics has a fixed fill tube. Furthermore, the wired Fecobionics contains a thin tube with four wires for power supply and data transmission (Fig. 1). The wires are connected to the USB port of a computer. Data are displayed in real time on the graphical user interface.

Along with the sensors, the probes contain a microprocessor that processes the data from the sensors before being transmitted by wires or wireless. In addition, the wireless Fecobionics contains batteries and a wireless radiofrequency transmitter (Fig. 1).

**Fig. 2.**
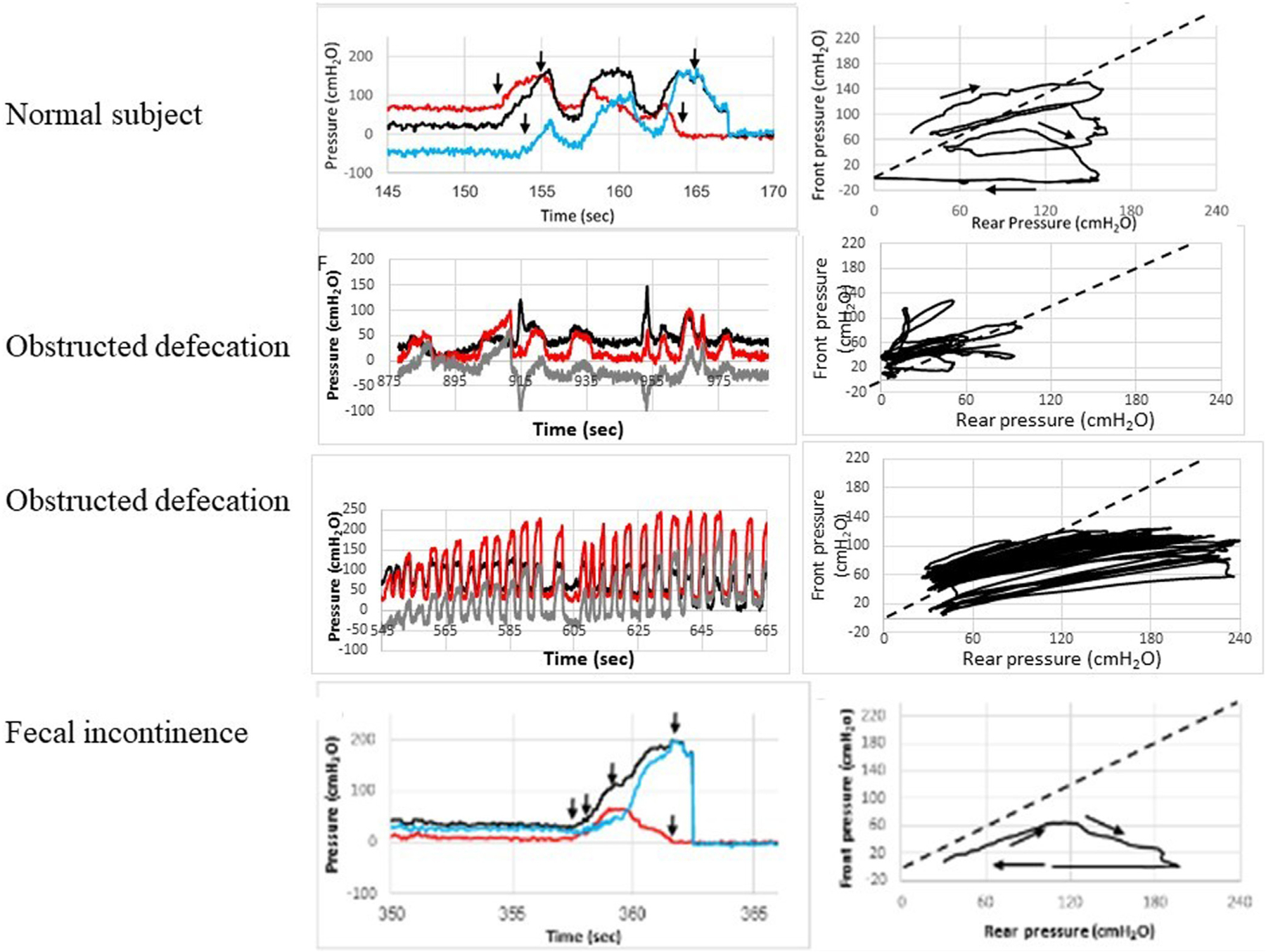
Pressure recordings of typical phenotypes from a normal subject, two patients with obstructed defecation, and a patient with fecal incontinence. **Left:** Front, rear, and front-rear pressure difference (the rectoanal pressure gradient) as function of time. Red, black and blue color indicate the front, rear and delta pressures. **Right:** Front pressure as function of rear pressure (preload-afterload diagram). The typical patterns are as follows: Normal subjects use 2–6 evacuatory contractions (in this case three contractions) that gradually move the tracings below the line of pressure unity (due to anal sphincter relaxation). Patients with obstructed defecation show uncoordinated (dyssynergic) patterns and often do not manage to evacuate the device. None of the two patients shown here evacuated the Fecobionics device. Fecal incontinence patients often have low anal pressure, reflected as the tracings being always below the line of pressure unity. Patients with fecal incontinence usually evacuate the device in one attempt and within a few seconds. In some patients with severe incontinence, the device may drop out unintendedly.

The integrated Fecobionics test provides opportunities for computing new parameters before and during evacuation along with the measured pressures, diameters, and anorectal angle. A novel approach to plot the data is the so-called preload-afterload diagram where the front pressure is plotted as a function of the rear pressure, i.e., resistive forces versus propulsive forces. The preload-afterload diagram has clearly contributed to our understanding of defecation patterns since phenotypes are different for normal subjects and patients with OD and FI (Fig. 2, [17,29]. Another relatively simple metric is the rectoanal pressure gradient (RAPG) that also weights the propulsive force against the resistive forces. RAPG is also used in HRAM studies but for unclear reasons the RAPG is negative during simulated defecation when it is supposed to be positive [8]. Such artifacts invalidate the use of the HRAM RAPG and makes it difficult for users to interpret the pathophysiological relevance. More advanced Fecobionics metrics have been developed including defecation indices (DIs) based on pressure differences, max pressures, volume, and duration of the defecation [29]. Defecation indices have proven valuable for subtyping patients with FI and OD. The defecation indices can be further developed and optimized, e.g., by taking into account the anorectal angle, anorectal diameters, and forces.

Patients with fecal incontinence usually evacuate the device in one attempt and within a few seconds. In some patients with severe incontinence, the device may drop out unintendedly.

### Procedures

At the inception of the wired probe, the focus was on the device evacuation, often preceded by procedures such as coughing to validate correct intrarectal placement of the device. Later, the approach shifted to imitate the London classification protocol for HRAM with standardized procedures such as coughs, anal squeezes, push, and straining [8]. The push procedure is an attempt to simulate defecation using HRAM. This is not needed, however, with Fecobionics which is evacuated, i.e., simulation not needed. Recently, it was realized that the London protocol is less useful for Fecobionics studies than originally thought. This is due to differences in technology between HRAM and Fecobionics. For example, the HRAM catheter straddles the anal canal and therefore obtains multiple measures of pressure along the sphincter. In contrast, Fecobionics is placed in rectum with the front located in the upper part of the anorectal junction. This explains why Fecobionics measures lower anal sphincter pressure while HRAM often uses the maximum pressure as the biomarker for function. The maximum pressure is most often recorded in the middle or distal part of the anal canal. Fecobionics measures the pressure at a high sampling rate during passage of the anal canal, but this is under conditions where the anal sphincter is somewhat relaxed due to the rectoanal inhibitory reflex (RAIR). To gain further insight in anal sphincter properties, anal pull-throughs are now included in the protocol where the subjects first relax and subsequently maximally contract the anal sphincter. Furthermore, push procedures (attempt to defecate by increasing abdominal pressure and relaxing the anal sphincter, i.e., a simulated defecation) were done in early trials but have now been eliminated because Fecobionics attempts real evacuation. It is important to know the distinction between simulated defecation (HRAM push procedure) and a simulated stool (Fecobionics).

Based on the above considerations, the current Fecobionics protocol, named the *Fecobionics San Diego Protocol*, encompass pull-throughs, coughs, anal squeezes, straining, bag filling and evacuation in privacy.

The entire study takes between 15 and 30 min depending on the specific protocol for selected patient groups. For example, most OD patients cannot evacuate the device in 2–3 min whereas normal subjects and FI patients usually evacuate it within 5–20 s. Most instructions to the patient are conveyed during the study and hence, does not require added time. The patients fill out fecal incontinence severity index (FISI) and constipation questionnaires before the Fecobionics study.

### Validation, safety studies and performance studies

Validation data on the technology have been published [24,27,54]. The studies have confirmed that measurements are accurate and valid including the electronic measurement of bending. Furthermore, safety data and information on adverse effects have been published from large animal and human studies [29,62]. No serious adverse effects have been recorded in more than 300 insertions in more than 180 human subjects.

New technologies must be compared to current technologies and new “normal ranges” must be provided if there are differences with existing technologies. In brief, Fecobionics have been shown to be consistent with BET on expulsion duration, despite the different filling volumes (BET is one-size-fits-all using 50 mL), and with defecography on anorectal angle measurements [33,36]. Unpublished data show that the anorectal angle at rest measured by Fecobionics correlates with the angles measured using defecography; *r* = 0.70, *P*<0.05) for the anterior wall of the rectum. The average anorectal angle for Fecobionics was 149°. For defecography, the average angle was 108°, 131°, and 152° measured at the posterior wall, mid-rectum, and anterior wall, respectively. This is reasonable since Fecobionics is positioned towards the anterior rectal wall. In contrast to BET and defecography, differences have been found between HRAM and Fecobionics, which can be explained by differences in protocols, device characteristics, device location, and experimental conditions [24]. Regardless, some degree of correlation exists between HRAM and Fecobionics [25,29]. Anal FLIP and Fecobionics have not been compared but differences are expected since Fecobionics measures during evacuation whereas FLIP makes distension in a fixed position in the anal canal.

The shape of Fecobionics visually fits the shape of feces type 3–4 on the BSFS. Although the BSFS is clearly refers to shape of feces, it is commonly believed to reflect consistency. Matsuda and coworkers studied the relation between the BSFS and consistency and found a clear association [44]. By testing the mechanical properties of the Fecobionics core and bag, it was found that Fecobionics had consistency corresponding to type 3–4 from the Matsuda paper (unpublished data).

**Fig. 3.**
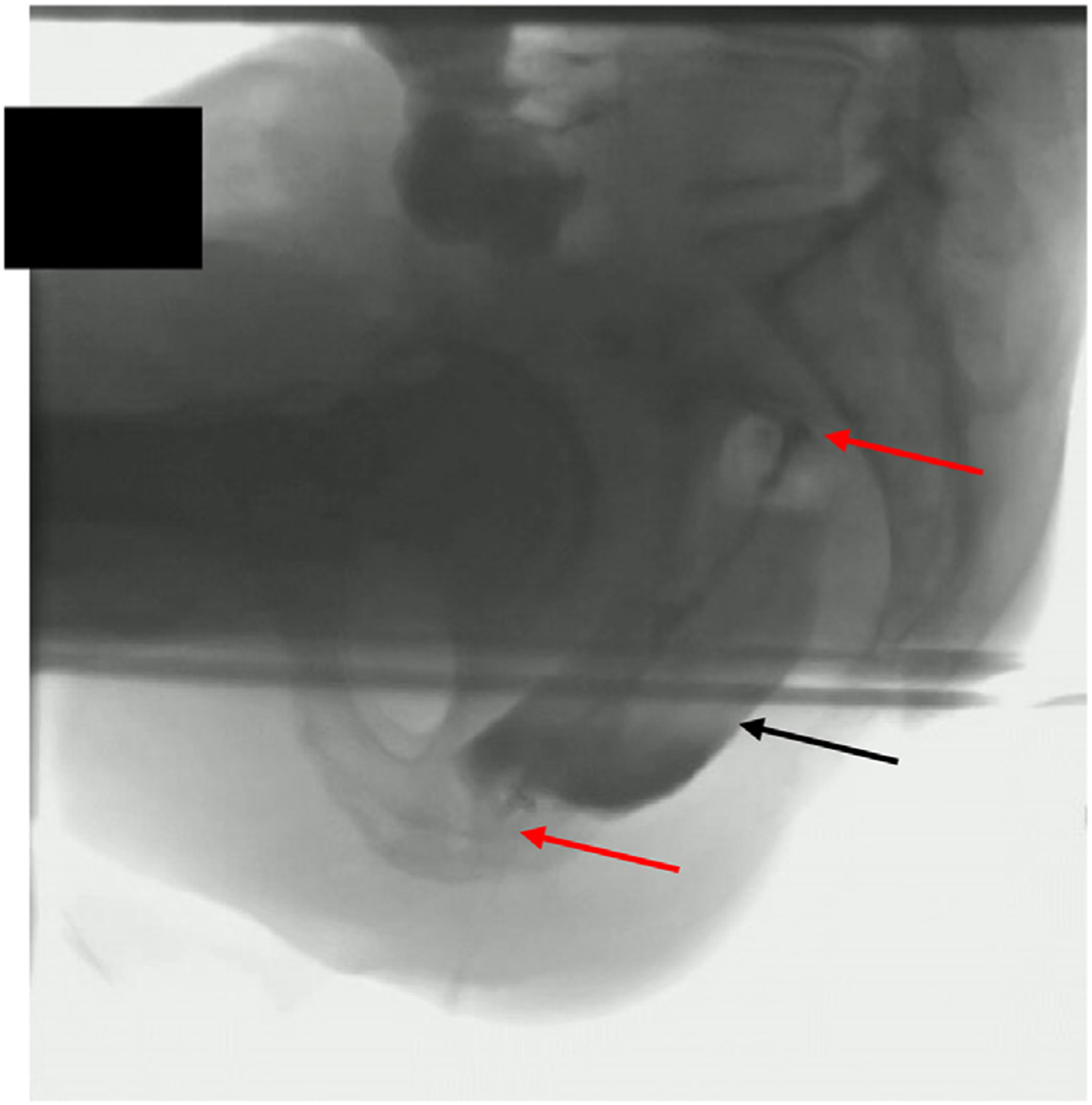
Defecographic image showing Fecobionics inside rectum shortly before it is being evacuated. The red arrows point to the front and rear of the 10cm-long Fecobionics device. The black arrow shows the bag filled with 80 ml of saline. Barium was injected into rectum before the Fecobionics device was inserted.

Many other validation data have been published such as combined Fecobionics-defecography for various purposes including confirming the location and bending (Fig. 3), angle measurements, and quantification of perineal descent [64]. Furthermore, test-retest repeatability has been tested and was found to be excellent for far most Fecobionics metrics [41].

Several performance studies have been published with Fecobionics on defecatory physiology [23,24,54,55]. Most attention has until now been given to pressure analysis in early studies using the wired Fecobionics device. Most normal subjects use only a few contractions to evacuate the Fecobionics device. It is quite commonly observed that normal subjects prepare for expulsion by going through several loop cycles with gradual front pressure decrease towards evacuation. Based on pressure-time plots, it is possible to divide defecations into five distinct phases based on the pressure patterns [27]. Rear-front pressure plots are an informative way to express Fecobionics pressure data. The rear-front pressure plot is inspired from the preload-afterload concept in cardiology, where left ventricle pressure-volume measurements provide substantial insights into heart contractility, preload (heart filling) and afterload (vascular resistance). This type of analysis is beneficial for understanding anorectal function. In cardiac physiology, although the preload is the degree of sarcomere stretch at the end of ventricular filling during diastole [20,58], end-diastolic volume and pressure are more practical for clinic measurements. Afterload is the pressure that the heart must work against to eject blood during systole. As aortic pressure increases, the afterload increases on the left ventricle. Since both the heart and GI have the function of transport, Fecobionics measurements enable the translation of pre-load/after-load concepts from cardiology to gastroenterology. The preloads correspond to the filling of the bag inside the rectum until the subject feels urge-to-defecate. Consequently, the subject initiates abdominal contractions to generate the propulsive force needed to expel the device. The propulsive (driving) force is measured by the rear pressure sensor, whereas the front pressure sensor records the afterload.

Fig. 2 shows representative pre-load/after-load defecations from normal subjects and patients with OD and FI. The diagram allows evaluation of pressure cycles without the time element (Fig. 2). For ease of interpretation, the line of unity is shown in the diagrams. When the front pressure exceeds the rear pressure, data are above the unity line (defecation cannot happen against a pressure gradient). The Fecobionics device (and feces) is expelled when the recto-anal pressure (force) gradient is large enough to overcome the friction between the surface and mucosa. Measurement of axial pressures at the front and rear, and the bag pressure is essential in this regard. Repeated contractions shift the tracings downwards where a cut-off is reached, i.e., the anal pressure drops quickly followed by device expulsion. Afterload is the resistance that the propulsive force must work against to evacuate feces. The resistance depends on several factors including the anal diameter, the anal pressure, anorectal angle, and friction. The diagrams in Fig. 2 show clockwise contraction cycles, for normal subjects usually 2–6 cycles, reflecting the number of abdominal muscle contractions that are needed to defecate. Fecobionics is uniquely designed to quantify these preload-afterload pressure properties where pressure-CSA relations and forces can be considered in the future. It is easily identifiable in the diagrams when anal relaxation occurs.

Although the preload-afterload diagrams are conceptionally very intuitive, they must be expressed into quantifiable metrics. This is doable as defecation indices computed as the areas under the front pressure curve (reflecting anal resistance) and rear pressure curve (reflecting propulsive force). The defecation indices can be normalized with respect to duration of the evacuation, maximum pressure amplitude, as well as other factors. Therefore, many defecation indices can be computed, and it remains to be determined which ones are most informative. Preliminary data have shown that abnormal subjects, who could not defecate BET and Fecobionics, showed abnormal values, especially for defecation indices that were not normalized for time. The afterload seems especially important since obstructed (dyssynergic) defecation [34,49,50] and anal stricture are associated with increased afterload. On the contrary, FI due to anal sphincter damage or impairment [48,63] are associated with decreased afterload. These properties are important for differentiating subtypes of patients. For example, the current dyssynergia classification [34,49,50] operates with a 2 × 2 diagram where two subtypes show abnormal expulsion pressures, and two subtypes are associated with anal sphincter function. The classification is criticized for being too simple. Furthermore, dyssynergic abnormality has been found in 90 % of healthy subjects with HRAM [24,32]. Interestingly, Fecobionics measurements showed abnormality in less than 25 % of normal subjects [24]. Fecobionics identified at least six subtypes of OD patients [25]. Due to increased afterload, the rectum (or abdominal muscles) must work harder to accomplish defecation. Long-term, this may lead to dyscoordination, hypertrophy, and altered rectal sensitivity [26]. Increased feces volume and deferred defecation may be associated with increased preload and afterload. These and other observations require further investigations.

Recently, Fecobionics was used to study the effect of posture on anorectal function [9]. The major finding was that it is very difficult to defecate in laying position whereas squatting and seated position did not differ significantly. Recent data suggest that Fecobionics can be used to detect sudden movements of the pelvic floor and perineal descent (Zhuoli., et al. 2021). This can be accomplished by analysis of the accelerometer data from the two MPUs in Fecobionics. In brief, data on acceleration was integrated to estimate velocity and integrated again to obtain change in position of Fecobionics. Good agreement was found between change in position during defecatory procedures estimated from the MPUs and from defecography.

**Fig. 4.**
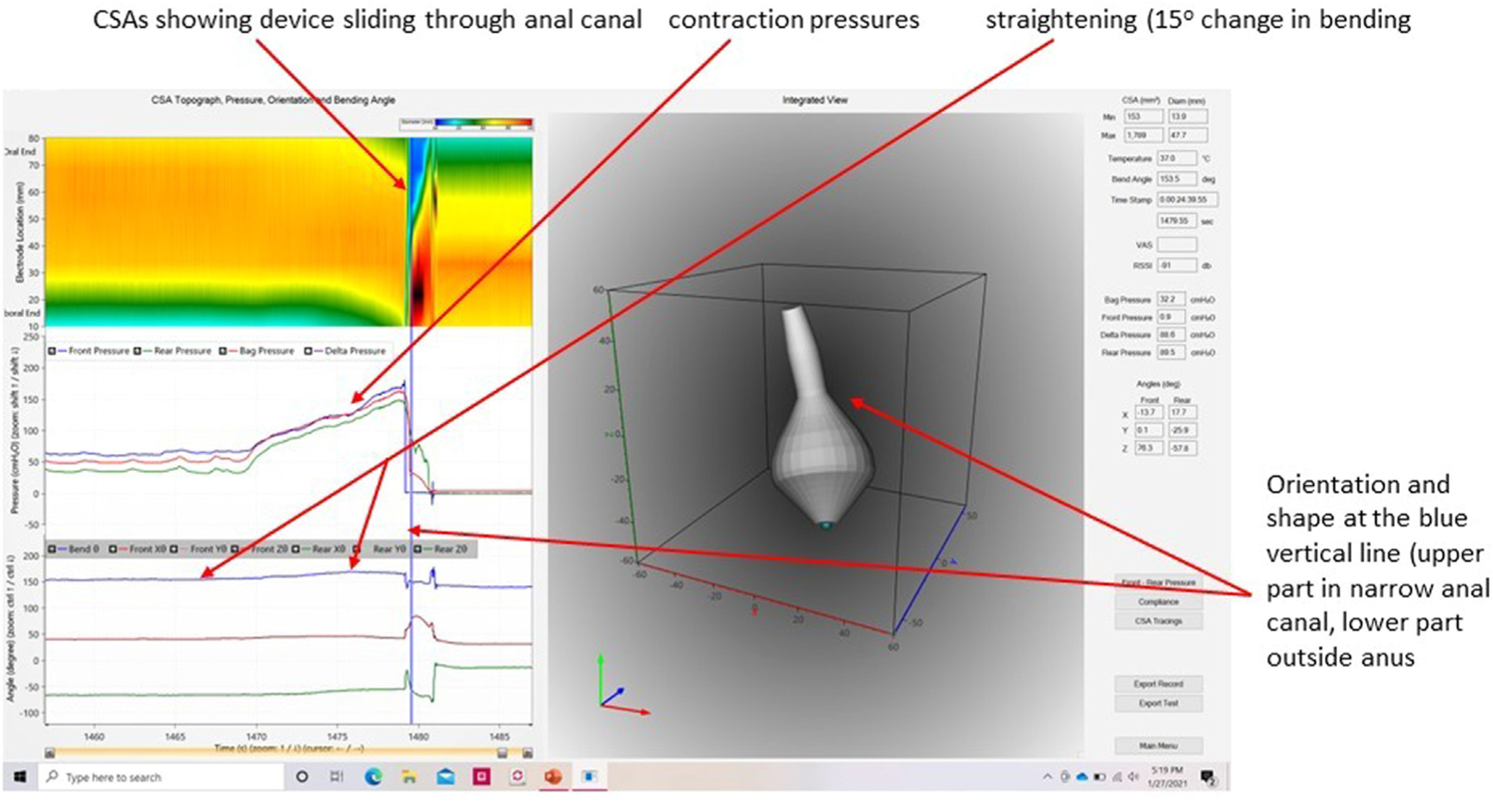
Graphical user interface showing data from a normal subject. **Left:** color contour of diameter data (top), pressures (middle) and bend angle and orientation of the front and rear relative to the direction of gravity. **Right:** Integrated view of the device geometry. The user interface shows 30 s of recording before, during and after the evacuation using just one defecatory contraction.

**Fig. 5.**
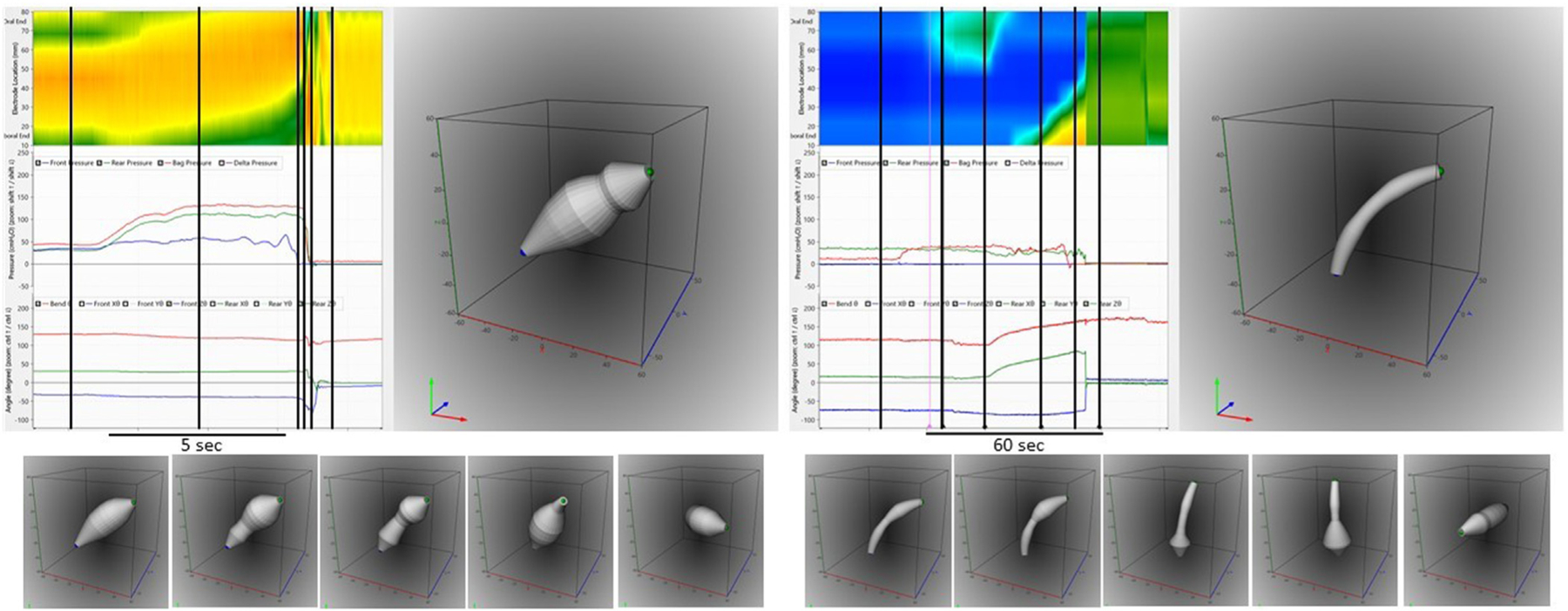
**Top:** The graphical user interface like the one shown in Fig. 4. **Left:** Data obtained in a normal subject after bag filling when the device is being evacuated. Right: Data obtained in a subject with severe fecal incontinence where the device drops out unintendedly during filling of the bag. The device geometry shown in the right figure for both subjects are obtained at the first black vertical line in the left diagrams. The device is located inside the rectum. **Bottom:** Geometry of the device at five selected time points before, during, and after the evacuation, corresponding to remaining five black vertical lines from each subject. The last subfigure for each subject shows the device when it has landed in the pot of the commode, and lays in a horizontal orientation. Evacuation pressures, rectal diameters, anal diameters including the minimum anal diameter, and the anorectal angle can be assessed from the data.

Two studies have been published on the new wireless probe on normal subjects where new metrics was presented. This included acceleration, velocity, contraction work and flow [29] Figs. 4 and 5 show images of the new graphical user interface where pressures, orientation, bending, and geometry are co-plotted. Naturally, the data are much better presented as video clips, e.g., at https://www.ncbi.nlm.nih.gov/pmc/articles/PMC9163794/

**Fig. 6.**
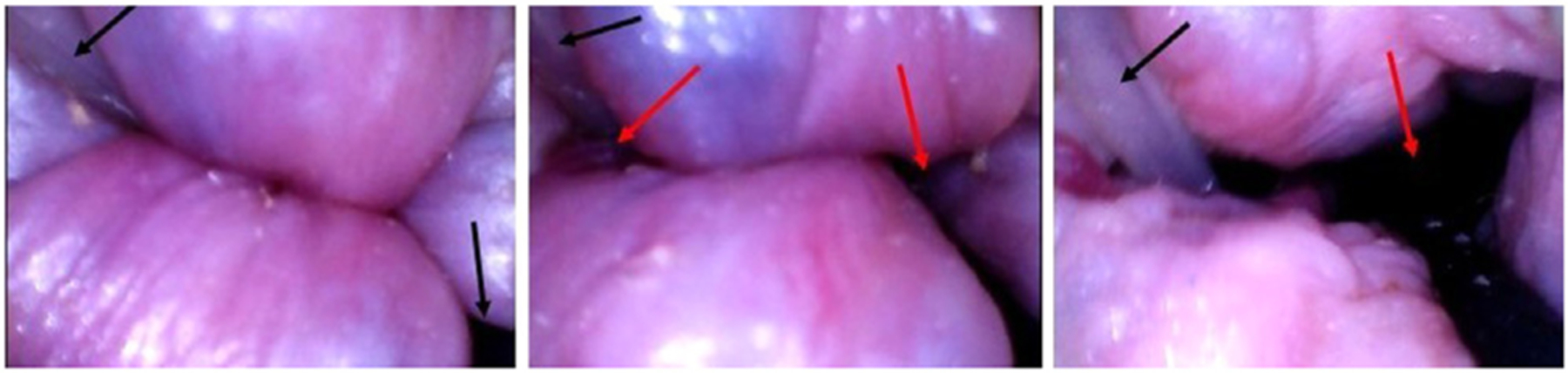
A series of images before and during initial evacuation of the Fecobionics device with video camera embedded at the front. The device was inserted into rectum for video imaging the anorectal junction. During the evacuation, the anal canal and exterior were visible. Mucosal folds (buckling), an internal hemorrhoid, an air passageway, and the fill tube are visible. The black arrows point to the connecting tubes. The red arrows show opening to outside anus.

Very recently, a study was published with a modified wired Fecobionics probe that contained a video camera at the front [53]. Hence this special Fecobionics probe combines endoscopic features with functional measurements. It was possible to view the anorectal junction and the anal canal during device evacuation (Fig. 6). As outlined in the section below on surgical perspectives, the applications are manifold due to the detailed information on surface anatomy of the anorectum.

### Clinical trials

Several clinical trials have been conducted or are currently being conducted. Finalized trials and publications are listed in Table 1.

Currently clinical trials are ongoing in the following areas:

- Deferred defecation and Fecobionics phenotypes.
- Comparison of Fecobionics and defecography in OD patients.
- Fecobionics-RAPG compared to HRAM-RAPG in FI and OD patients.
- Detection of air passageways in FI patients.
- LARS phenotypes and their correlation to symptoms.
- Evaluation of the mechanisms and the effect of sacral nerve stimulation.
- FI and biofeedback therapy (new Fecobionics probe on larger scale cohort).
- Obstructed defecation (new Fecobionics probe on larger scale cohort).
- Monitoring of biofeedback therapy in OD patients.

### Perspectives in gastroenterology, surgery and pediatrics

Fecobionics technology is wholistic due to the integration of several technologies. It is a simulated stool during actual expulsion that enables calculation of new metrics and computer modeling of defecation. The biomechanical analysis can enhance our physiological understanding of defecation and future interdisciplinary research for unraveling GI transit, defecatory function, anorectal sensory-motor disorders, and symptoms. This is a step in the direction of improved diagnosis and therapy of anorectal diseases. The disposable Fecobionics device can be produced at a cost below the US reimbursement rate for functional anorectal procedures. The various application areas of Fecobionics include the following:

- Physiological assessment
- Diagnostics
- Monitoring of therapy
- Fecobionics-based biofeedback therapy

Studies have been published on physiological assessment, diagnostics, and monitoring of biofeedback therapy in patients with fecal incontinence [17]. Needless to say, much more clinical research must be done. A major goal is that Fecobionics can serve as a tool for biofeedback therapy. The graphical user interface facilitates new ways of displaying the anorectal muscle actions for the patients who can do pelvic floor exercises and see directly how it affect different muscle groups and the defecatory function on the monitor. The graphical user interface can inform the patients about correctly or incorrectly performed maneuvers. This will be a major focus in the coming years in the belief that biofeedback therapy can be improved and occur closer to the point-of-care, even in the home of patients with or without remote guidance by healthcare personnel.

In addition to design changes of the graphical user interface for biofeedback therapy, future physiological and clinical studies may require probe design diversity. A Fecobionics prototype with a video camera has been developed and will soon be trialed. Other types of diversified probes can be used for constipation associated with hard stools. Since these patients may be able to defecate the current soft Fecobionics, devices with higher stiffness may be developed. This can be accomplished by using a different resin to construct the core or using a gel-like fluid in the bag with higher viscosity. Furthermore, other sensors such as emg electrodes can be implemented in the device. Fecobionics prototypes can also be designed for use in other organs. In fact, a device with an external battery package has already been developed for studying colon function in canines [23]. A better understanding of colonic physiology is important for understanding lower GI tract functional disorders such as slow transit constipation. Fecobionics was able to measure shallow antegrade and retrograde contractions in the canine colon as variations in cross-sectional areas [28]. The shallow contractions are believed to serve an important function for slowing down colonic transit and facilitate mixing of fecal content. Translating the technology to humans with colonoscope insertion of Fecobionics will be the next major step. Furthermore, efforts are being made to construct a swallowable device that can provide important information on esophagus function including simultaneous pressures, acceleration, velocity, position, and orientation [40]. This may be useful in the diagnosis of achalasia and esophagogastric junction outlet obstruction (EGJOO).

### Surgical aspects

Several anorectal diseases may require surgical intervention. This spans over a large range of surgeries including for hemorrhoids, rectocele, full thickness rectal prolapse, rectal intussusception, enterocele, rectal cancer, et cetera. For example, OD can be associated with significant findings at defecography indicating structural blockage (rectal invagination, intussusception, enterocele or rectocele). However, even perfect surgical correction of the presumed underlying cause may not lead to functional improvement of OD, indicating that OD often is a physiological disease. Therefore, it is of predictive value to further subtype rectal diseases before surgical repair. In addition to functional measurements, it is anticipated Fecobionics with video camera can be used to guide repair surgery by visualizing the anorectal junction from rectum during the procedure.

LARS is a result of surgery for low rectal cancer and unfortunately affects up to 75 % of patients undergoing this procedure. It is a condition that is not fully understood. One study has been published showing severe phenotypes in LARS patients [11]. Clearly, there is an unmet need for further studies to understand this condition in patients.

Sacral nerve stimulation (SNS) is a technology where a stimulator is implanted by the surgeon for stimulating the nerves supplying anorectum. SNS is indicated for patients with severe FI where other treatments have been unsuccessful. In general, the mechanism of SNS is not known as studies have pointed to effects on the anal muscles, rectal sensitivity [15] and increased retrograde colonic motility. It is believed that Fecobionics can shed light on SNS mechanisms since those mechanisms likely affect the preload-afterload properties.

### Pediatric aspects

Children have the same basic anorectal anatomy as adults. Anorectal disorders also appear the same in children as in adults with constipation and FI being common in both adults and children. A meta study reported the prevalence of functional constipation in children ranging from 1 to 30 %, with a pooled prevalence of 9.5 % (95 % CI 7.5–12.1) [37,60]. It is the principal complaint in 3–5 % of all pediatric outpatient clinics and as many as 35 % of all visits to pediatric gastroenterologists [6]. However, there are clear differences between children and adults, e.g., in constipation, pediatric patients are often boys, have a withholding pattern, and more often have mixed OD-FI whereas in adults, they are mainly women, have a straining pattern, and FI is less common [14]. The effect of biofeedback therapy is unclear in children but established for dyssynergic defecation in adults. Biofeedback therapy in pediatrics can be improved and needs further study with new tools.

Fecobionics has until now been used in adults only, but some studies included Asians weighing as low as 40–45 kg. This weight corresponds to 12-year-old kids from the West. For younger or smaller children, the probe can be miniaturized, e.g., it has been possible to produce a device that is 5cm-long and 7 mm diameter with full functionality. Since this size is smaller than typical feces in 6–11 years old kids, there would not be specific safety concerns for studies in younger kids. However, children have a different perception and may not be comfortable with rectal insertions. This is also the case for other anorectal test technologies. On the other hand, many children are good at interpreting graphics and enjoy playing computer games, which may provide an advantage for the Fecobionics graphical user interface, that can be modified for biofeedback therapy in different age groups to teach the kids how to control pelvic floor muscles using animations familiar to different age groups.

## Conclusion

In summary, Fecobionics is a novel disruptive technology in its infancy that has substantial potential for translating anorectal assessment of function. Successful application of Fecobionics has been demonstrated in animals and humans. It is the first intraluminal device that can measure a variety of physiological variables during evacuation. Fecobionics provides assessment of a range of biomarkers that will be important in research and clinical practice. The clinical future of Fecobionics ultimately depends on its ability to change the management of patients with anorectal disorders.

## Figures and Tables

**Table 1:** List of published clinical trials.

Authors	Topic/disorder	Major clinical results and correlation with symptoms
Gregersen et al 2020 [23] and Futaba et al 2022 [18]	Fecal incontinence	Simple HRAM measures do not differentiate patients from normal subjects. Fecobionics defecation indices showed difference. Fecobionics showed higher correlation to symptoms as compared to HRAM-BET.
Gregersen et al 2021 [25]	Obstructed defecation, OD	Six OD phenotypes were identified using Fecobionics. The ARM-BET data differed significantly from Fecobionics data. Correlation between tests was poor with the R^2^ between 0.08 and 0.30.
Chen et al 2022 [11]	Low anterior resection syndrome	LARS patients have severely impaired defecatory function and more severe FI symptoms than the control FI patient group. The impairment is caused by dysfunctional anal sphincter, low rectal volume, and dyscoordination. Fecobionics revealed parameters that provide more detailed analysis of the dysfunction compared to conventional technologies and correlated better with symptoms than ARM-BET.
Futaba et al 2022 [17]	Monitoring biofeedback therapy for fecal incontinence	Fecobionics predicted the outcome of biofeedback therapy. Fecobionics demonstrated a better association with FISI scores than current technologies.
Gregersen and Mittal 2023 [31]	Rectal contraction in patients with fecal incontinence and obstructed defecation	Rectal contractility differs between normal subjects, and patients with OD and FI. Association between measured data and symptoms not analyzed specifically.
